# Supplementary material for: Resolving the full spectrum of human genome variation using Linked-Reads
Source: Genome Res. 2019 Apr;29(4):635–45. doi: 10.1101/gr.234443.118 (PMC6442396; doi:10.1101/gr.234443.118)
Supplement: Supplemental Material [file supp_gr.234443.118_Supplemental_longranger-2.2.zip › longranger-2.2.tar.bz2!/lr2.2-public/lib/stan/cmdstan/stan/doc/api/html/functions_eval.html]

Stan: Class Members - Enumerator


|  |  |
| --- | --- |
|  | Stan  2.5.0  probability, sampling & optimization |


- Main Page
- Related Pages
- Namespaces
- Classes
- Files

- Class List
- Class Index
- Class Hierarchy
- Class Members

- All
- Functions
- Variables
- Typedefs
- Enumerator
- Related Functions

- a
- c
- d
- h
- i
- l
- m
- n
- o
- r
- s
- u
- v


All Classes Namespaces Files Functions Variables Typedefs Enumerations Enumerator Friends Macros Pages

### - a -

- AddCost
  : Eigen::NumTraits< stan::agrad::var >

### - c -

- CONFIG
  : stan::gm::error\_codes

### - d -

- DATAERR
  : stan::gm::error\_codes

### - h -

- HasFloatingPoint
  : Eigen::NumTraits< stan::agrad::var >

### - i -

- IsComplex
  : Eigen::NumTraits< stan::agrad::var >
- IsInteger
  : Eigen::NumTraits< stan::agrad::var >
- IsSigned
  : Eigen::NumTraits< stan::agrad::var >

### - l -

- LhsStorageOrder
  : Eigen::internal::general\_matrix\_vector\_product< Index, stan::agrad::var, ColMajor, ConjugateLhs, stan::agrad::var, ConjugateRhs >
  , Eigen::internal::general\_matrix\_vector\_product< Index, stan::agrad::var, RowMajor, ConjugateLhs, stan::agrad::var, ConjugateRhs >

### - m -

- MulCost
  : Eigen::NumTraits< stan::agrad::var >

### - n -

- NOINPUT
  : stan::gm::error\_codes

### - o -

- OK
  : stan::gm::error\_codes

### - r -

- ReadCost
  : Eigen::NumTraits< stan::agrad::var >
- RequireInitialization
  : Eigen::NumTraits< stan::agrad::var >

### - s -

- SOFTWARE
  : stan::gm::error\_codes

### - u -

- USAGE
  : stan::gm::error\_codes

### - v -

- value
  : stan::contains\_fvar< T1, T2, T3, T4, T5, T6 >
  , stan::error\_index
  , stan::is\_constant< T >
  , stan::is\_constant\_struct< T >
  , stan::is\_constant\_struct< Eigen::Block< T > >
  , stan::is\_constant\_struct< Eigen::Matrix< T, R, C > >
  , stan::is\_constant\_struct< std::vector< T > >
  , stan::is\_fvar< T >
  , stan::is\_fvar< stan::agrad::fvar< T > >
  , stan::is\_var< T >
  , stan::is\_var< stan::agrad::var >
  , stan::is\_var\_or\_arithmetic< T1, T2, T3, T4, T5, T6 >
  , stan::is\_vector< T >
  , stan::is\_vector< const T >
  , stan::is\_vector< Eigen::Block< T > >
  , stan::is\_vector< Eigen::Matrix< T, 1, Eigen::Dynamic > >
  , stan::is\_vector< Eigen::Matrix< T, Eigen::Dynamic, 1 > >
  , stan::is\_vector< std::vector< T > >
  , stan::is\_vector\_like< T >
  , stan::is\_vector\_like< const T >
  , stan::is\_vector\_like< Eigen::Matrix< T, Eigen::Dynamic, Eigen::Dynamic > >
  , stan::is\_vector\_like< T \* >
  , stan::prob::include\_summand< propto, T1, T2, T3, T4, T5, T6, T7, T8, T9, T10 >


---

[ Stan Home Page ]

*© 2011–2014,
Stan Development Team.*
